# Evaluation of building washing machines as an extreme environment for potentially pathogenic fungi

**DOI:** 10.1038/s41598-023-48598-0

**Published:** 2023-12-04

**Authors:** Zahra Zareshahrabadi, Neda Amirzadeh, Keyvan Pakshir, Reza Nasr, Rasoul Mohammadi, Kamiar Zomorodian

**Affiliations:** 1 Basic Sciences in Infectious Diseases Research Center, Shiraz University of Medical Sciences, Shiraz, Iran; 2 Department of Medical Parasitology and Mycology, School of Medicine, Shiraz University of Medical Sciences, Shiraz, Iran; 3 Department of Medical Parasitology and Mycology, School of Medicine, Infectious Diseases and Tropical Medicine Research Center, Isfahan University of Medical Sciences, Isfahan, Iran

**Keywords:** Microbiology, Molecular biology, Environmental sciences, Diseases, Health care

## Abstract

Washing machines are commonly used in households and are considered indispensable appliances for maintaining cleanliness and hygiene. Environmental conditions within household washing machines are ideal for fungal colonization, which may pose risks to human health and contribute to sick building syndrome. This study aimed to investigate the fungal species contamination in the building washing machines. A total of 50 building washing machines were swab-sampled at three locations: the detergent drawer, the inner and outer parts of the rubber door seal. The housekeeping conditions of these appliances were assessed through a questionnaire. The isolated fungi were identified using standard mycological diagnostic procedures and molecular analysis based on the ITS1/ITS4 and β-tubulin gene regions. The possibility of fungal agents transferring from contaminated washing machines to autoclaved clothes during laundry cycles was investigated. Fungi were detected in 82% of the sampled appliances, with the inner rubber door seal being the most frequently colonized area. Using conventional and molecular techniques, we identified 122 fungal isolates, encompassing 17 diverse genera of molds, yeast-like, and yeast fungi. The mold fungi included 14 genera of hyaline and black genus. Among these, the most frequently identified genera of hyaline and black fungi were *Aspergillus* (27.7%), and *Cladosporium* (10.7%), respectively. This study demonstrates that building washing machines may serve as suitable ecological niches for fungal growth and transmission. Therefore, regular cleaning and disinfection of these devices are necessary.

## Introduction

The microbial content of airborne particles, both indoors and outdoors, is of paramount concern from both environmental and public health perspectives. Sick building syndrome (SBS) is a term used to describe a condition in which occupants of a building experience illness symptoms due to indoor air pollution^1^. Variable SBS symptoms encompass headaches, vertigo, irritation of the eyes, nose, and esophagus, and as well as respiratory complications. One potential source of indoor air pollution contributing to SBS is fungal contamination. Fungi have the potential to adversely affect health and well-being, particularly through the allergenicity of fungal elements and the presence of toxic secondary metabolites^2–5^. Numerous fungal species can colonize indoor environments within buildings, including walls, furniture, and household appliances such as washing machines (WMs) and dishwashers, subsequently producing spores and volatile organic metabolites^5^. These building WMs, commonly found in laundry facilities in buildings, are an area of interest due to their potential as a niche for the growth and dissemination of fungi. These fungal spores and microbial compounds can lead to irritation, respiratory illnesses, cytotoxicity, fatigue, allergic reactions, asthma, and infections, especially in immunocompromised individuals^6,7^.

Fungi can colonize both visible and hidden areas of WMs under favorable micro-environmental conditions, such as reduced washing temperatures, moisture, and the use of biodegradable detergents, leading to fungal enrichment and biofilm formation^8,9^. Importantly, microbial biofilms may serve as reservoirs of pathogenic microorganisms that can contaminate laundry, potentially endangering the health of susceptible individuals. Previous research has demonstrated that WMs can be colonized by a wide variety of microorganisms and often develop biofilm structures on their surfaces^10^. Using molecular methods, Nix et al.^11^ investigated microorganisms at various sites within WMs and identified representatives of the Basidiomycota and Ascomycota as the predominant fungal colonizers in these appliances.

In the context of household appliances, previous studies have emphasized the importance of cleaning performance and energy efficiency during laundering processes, with less emphasis on the hygienic impact of WMs^4,9,12^. Several studies have suggested that fabrics and textiles can play a role in the transmission of infections, demonstrating the potential for microbial respiratory infection spread associated with WM usage during laundering^1,2,12^. Fungi residing in WMs can potentially disseminate into the kitchen or any environment where the WM is located through aerosols, wastewater, and direct contact between contaminated WMs and users’ hands^7,13^.

Furthermore, there is evidence linking fungal contamination in WMs to indoor air pollution, which has been associated with SBS. Consequently, WMs may serve as a source of environmental fungal contamination and its associated effects^14,15^. The favorable conditions within WMs, characterized by moisture, humidity, and nutrient-rich environments, promote fungal growth, biodeterioration, and discoloration. These machines are subjected to fluctuating temperature and humidity levels, often harboring residual moisture that can persist over time. Furthermore, the organic materials frequently encountered in WMs, such as lint and detergent residues, can serve as substrates for fungal growth^4,9,15^. Given these conditions, it is conceivable that fungi could colonize and persist within building WMs, posing potential risks to residents, particularly those with compromised immune systems. As a result, this poses hygiene risks, diminishes the aesthetic appeal of WMs, and shortens their useful lifespan^7,11^. Current research focuses on detecting emerging fungi in indoor environments, whose diversity and potency may differ from those in natural environments. While these investigations have provided valuable insights into indoor fungal ecology, the unique conditions within WMs have received limited attention. The primary objective of this study was to assess fungal diversity within the specific environment of building WMs, with due consideration of potential risks to users. We also aimed to assess the potential transfer of fungal agents from contaminated washing machines to clean cloths.

## Result

### Building WMs usage parameters and characterization

In this investigation, we employed a questionnaire to gather data on various usage parameters related to WMs within the studied environment (Table 1). The age range for the 82% of WMs that were positive for fungi growth was 6–10 years. This observation suggests a potential relationship between the age of the machines and the likelihood of fungal contamination. Older machines may develop wear and tear that could create suitable conditions for fungal colonization.

**Table 1:** Relationship between the fungal pollution and the questionnaire data of the studied WMs.

Variables	Fungal contaminated	Uncontaminated	Total
Loading type			
Front	38	9	47 (94%)
Top	3	0	3 (6%)
Age			
1–5 years	12	5	17 (34%)
6–10 years	18	2	20 (40%)
> 10 years	11	2	13 (26%)
WMs location			
Kitchen	31	7	38 (76%)
Balcony	6	0	6 (12%)
Laundry room	4	2	6 (12%)
Ventilation type			
Window	12	3	15 (30%)
Kitchen fan	7	1	8 (16%)
Kitchen hood	11	4	15 (30%)
Fan & hood	1	0	1 (2%)
Window & hood	1	0	1 (2%)
Window & fan	3	1	4 (8%)
Fresh air	6	0	6 (12%)
Laundry time			
Weekly 1–2	21	6	27 (54%)
Weekly 3–4	13	4	17 (34%)
Weekly 5–7	5	0	5 (10%)
Monthly	1	0	1 (2%)
Wash time			
10–30 min	6	0	6 (12%)
30–60 min	14	6	20 (40%)
> 1 h	21	3	24 (48%)
Washing temperature			
30 ℃	19	4	23 (46%)
40 ℃	12	2	14 (28%)
≥ 60 °C	3	1	4 (8%)
Variable	7	2	9 (18%)
Laundry detergent			
Powder	21	5	26 (52%)
Liquid	11	1	12 (24%)
Both	9	3	12 (24%)
Laundry softener			
With softener	17	4	21 (42%)
Without softener	24	5	29 (58%)
Laundry bleach			
With bleach	12	3	15 (30%)
Without bleach	29	6	35 (70%)
Drying rubber door seal			
Drying with towel	3	2	5 (10%)
Drying with air flow	29	7	36 (72%)
Without drying	9	0	9 (18%)
Pigmentation			
With pigmentation	14	0	14 (28%)
Without pigmentation	27	9	36 (72%)
Pigmentation color			
Dark	9	0	9 (64%)
Yellow	5	0	5 (36%)
Pigmentation site			
Powder drawer	9	0	9 (60%)
Door rubber	4	0	4 (27%)
Powder drawer & door rubber	2	0	2 (13%)
Building type			
Apartment	29	8	37 (74%)
Traditional house	12	1	13 (26%)
Building age			
< 5 years	15	3	18 (36%)
5–10 years	10	3	13 (26%)
> 10 years	16	3	19 (38%)
Building ventilation			
Water cooler	33	8	41 (82%)
Air conditioner	6	0	6 (12%)
Water cooler & Air conditioner	2	1	3 (6%)
House resident numbers			
1–2	10	5	15 (30%)
3–4	26	4	30 (60%)
5–6	4	0	4 (8%)
≥ 7	1	0	1 (2%)
Cleaning WMs routine			
Weekly cleaning	3	0	3 (6%)
Monthly cleaning	1	2	3 (6%)
Without cleaning	37	7	44 (88%)
Total WMs	41 (82%)	9 (18%)	50 (100%)

The most commonly observed washing temperature associated with favorable fungal growth was 30°C. This finding is of importance, as different temperatures can impact the survival and growth of fungi. Notably, our study revealed that 31% of the contaminated WMs were situated in kitchens, raising concerns about the presence of fungi in an area crucial for food preparation and hygiene. Additionally, our research highlighted that a significant portion (37%) of contaminated WMs were not subject to a regular cleaning routine. However, as shown in Table 1, our statistical analysis did not find any statistically significant correlations (*p*-values > 0.05) between fungal growth and the studied variables (*p*-values > 0.05).

### Conventional and molecular identification of fungal isolates

**Figure 1:**
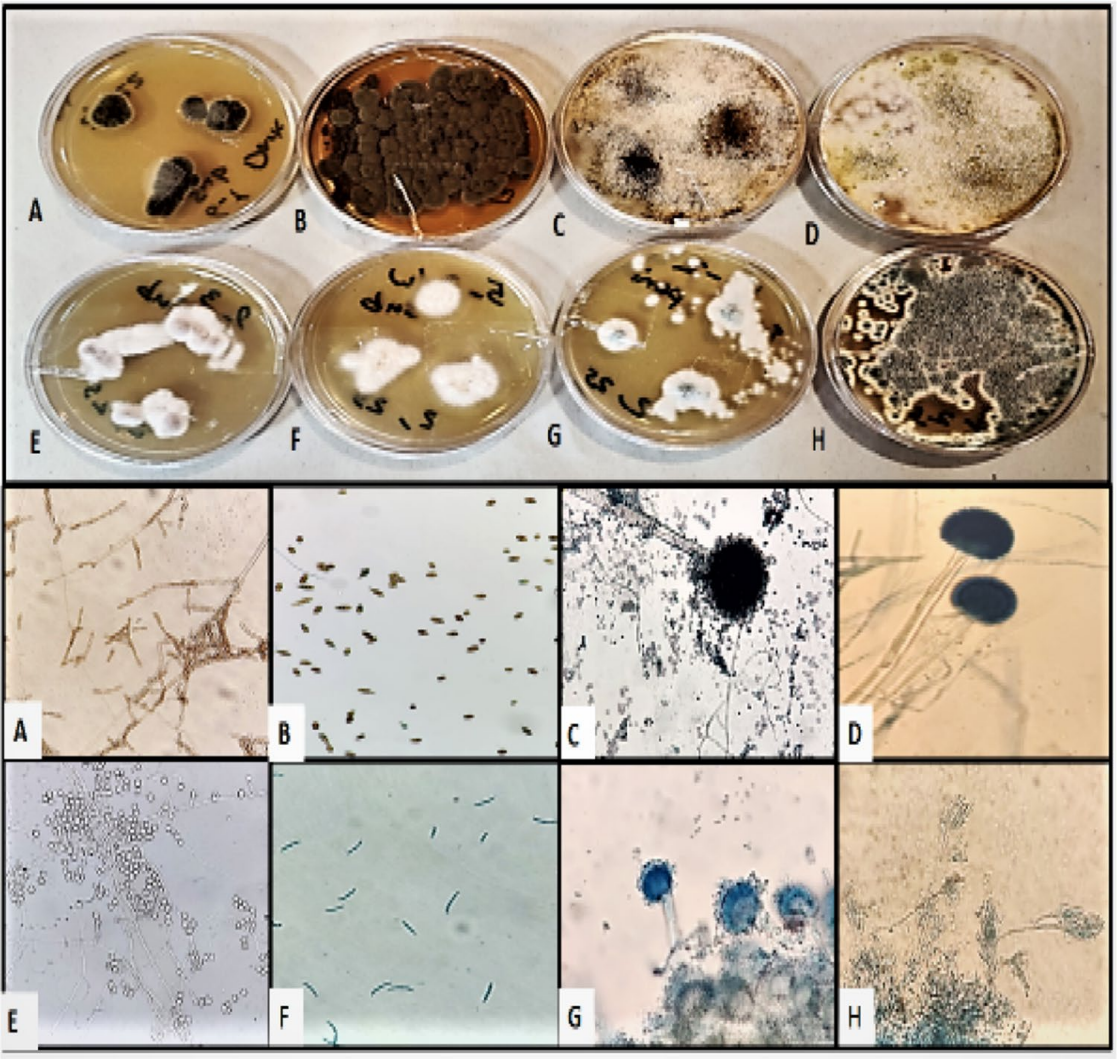
Morphological identification of fungi assessed by using teas mounts and slide culture methods.

**Figure 2:**
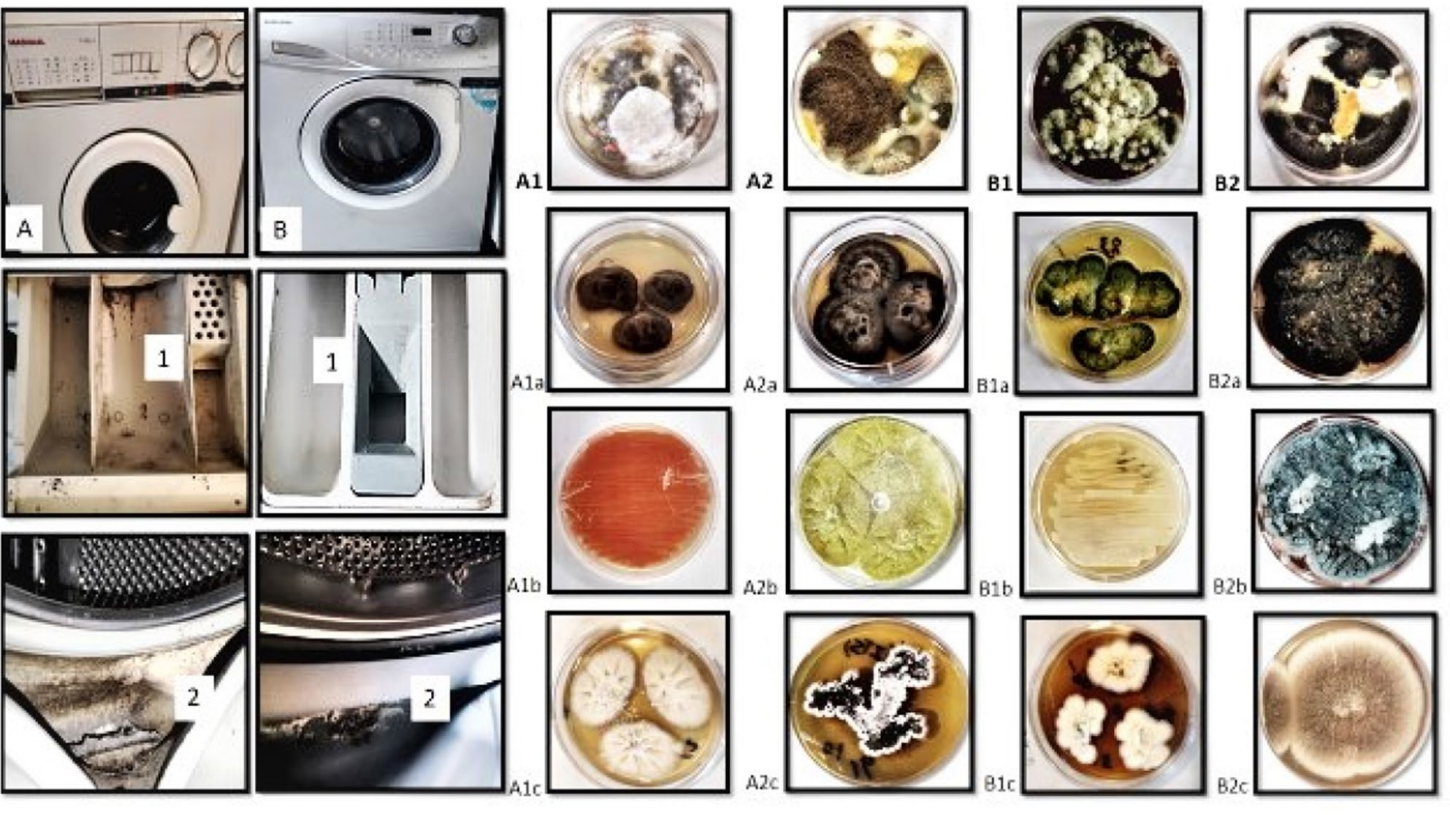
(**A**) (1, 2) & (**B**) (1, 2): Sampling sites in the WMs. A1, A2, B1, and B2: Primary isolation of different fungi from WMs swab samples. Molecular identification of fungal isolates; A1a: *Ochroconis musae*, A1b: *Rhodotorula* spp., A1C: *Chaetomium madrasense*, A2a: *Exophiala mesophila*, A2b: *Aspergillus flavus*, A2c: *Scopulariopsis gracilis*, B1a: *Aspergillus multicolor*, B1b: *Candida parapsilosis*, B1c: *Fusarium prolifratum*, B2a: *Alternaria sorghi*, B2b: *Penicillium chrysogenum*, B2c: *Aspergillus terreus.*

Out of the 50 building WMs tested, 41 (82%) were found to be positive for fungal contamination. The inner parts of the rubber door seals exhibited the highest contamination rate (37.5%), followed by the outer parts of rubber door seals (36.1%), and detergent drawers (26.2%) (Figs. 1 and 2). Among the 122 fungal species isolated, 17 genera of molds, yeast-like fungi, and yeasts were identified. Sequencing revealed that the mold fungi consisted of seven genera of hyaline molds and seven genera of black mold fungi at various species levels (Tables 2 and 3). Notably, *Aspergillus* spp. (27.7%), *Penicillium* spp. (11.4%), *Fusarium* spp. (4.8%), *Scopulariopsis* spp. (2.4%), *Acremonium* spp. (1.6%), *Mucor* spp. (1.6%), and *Corallomycetella* sp. (0.8%) are listed in Table 2 as the hyaline fungi isolated in this investigation.

**Table 2:** Frequency of hyaline fungi isolated from building WMs.

	Genus	Species	Number	Genus	Species	Number
Hyaline molds	*Aspergillus* 34 (27.7%)	*Aspergillus niger*	13 (10.7%)	*Fusarium* 6 (4.8%)	*Fusarium oxysporum*	2 (1.6%)
		*Aspergillus flavus*	11 (9%)		*Fusarium solani*	1 (0.8%)
		*Aspergillus fumigatus*	2 (1.6%)		*Fusarium prolifratum*	3 (2.4%)
		*Aspergillus terreus*	2 (1.6%)			
		*Aspergillus tubingensis*	1 (0.8%)	*Scopulariopsis* 3 (2.4%)	*Scopulariopsis gracilis*	1 (0.8%)
		*Aspergillus calidoustus*	1 (0.8%)		*Scopulariopsis brumptii*	2 (1.6%)
		*Aspergillus sydowii*	1 (0.8%)			
		*Aspergillus montevidensis*	1 (0.8%)	*Acremonium* 2 (1.6%)	*Acremonium implicatum*	1 (0.8%)
		*Aspergillus protuberus*	1 (0.8%)		*Acremonium incoloratum*	1 (0.8%)
		*Aspergillus multicolor*	1 (0.8%)	*Mucor* 2 (1.6%)	*Mucor*	2 (1.6%)
				*Pseudallescheria* 1 (0.8%)	*Pseudallescheria boydii*	1 (0.8%)
	*Penicillium* 14 (11.4%)	*Penicillium chrysogenum*	2 (1.6%)	*Corallomycetella* 1 (0.8%)	*Corallomycetella repens*	1 (0.8%)
		*Penicillium brevicompactum*	1 (0.8%)			
		*Penicillium sp*	11 (9%)

**Table 3:** Frequency of black molds and yeasts isolated from building WMs.

	Genus	Species	Number		Genus	Species	Number
Black molds	*Cladosporium* 13 (10.7%)	*Cladosporium halotolerans*	6 (2.5%)	Black molds	*Chaetomium* 2 (1.6%)	*Chaetomium madrasense*	1 (0.8%)
		*Cladosporium macrocarpum*	3 (0.8%)			*Chaetomium elatum*	1 (0.8%)
		*Cladosporium cladosporioides*	3 (0.8%)		*Stemohylium* 2 (1.6%)	*Stemphylium spp.*	2 (1.6%)
		*Cladosporium sp.*	1 (6.6%)		*Phoma* 1 (0.8%)	*Phoma herbarum*	1 (0.8%)
	*Ochroconis* 11 (9%)	*Ochroconis musae*	6 (4.9%)		*Curvularia* 1 (0.8%)	*Curvularia Sp*	1 (0.8%)
		*Ochroconis mirabilis*	2 (1.6%)	Yeast	*Rhodotorula* 15 (12.3%)	*Rhodotorula spp.*	15 (12.3%)
		*Ochroconis constricta*	2 (1.6%)		*Candida* 8 (6.6%)	*Candida parapsilosis*	6 (4.8%)
		*Ochroconis tshawytschae*	1 (0.8%)			*Candida orthopsilosis*	1 (0.8%)
	*Exophiala* 3 (2.5%)	*Exophiala mesophila*	3 (2.5%)			*Candida lusitaniae*	1 (0.8%)
	*Alternaria* 2 (1.6%)	*Alternaria sorghi*	1 (0.8%)	Yeast-like	*Geotrichum* 1 (0.8%)	*Geotrichum candidum*	1 (0.8%)
		*Alternaria porri*	1 (0.8%)				

**Figure 3:**
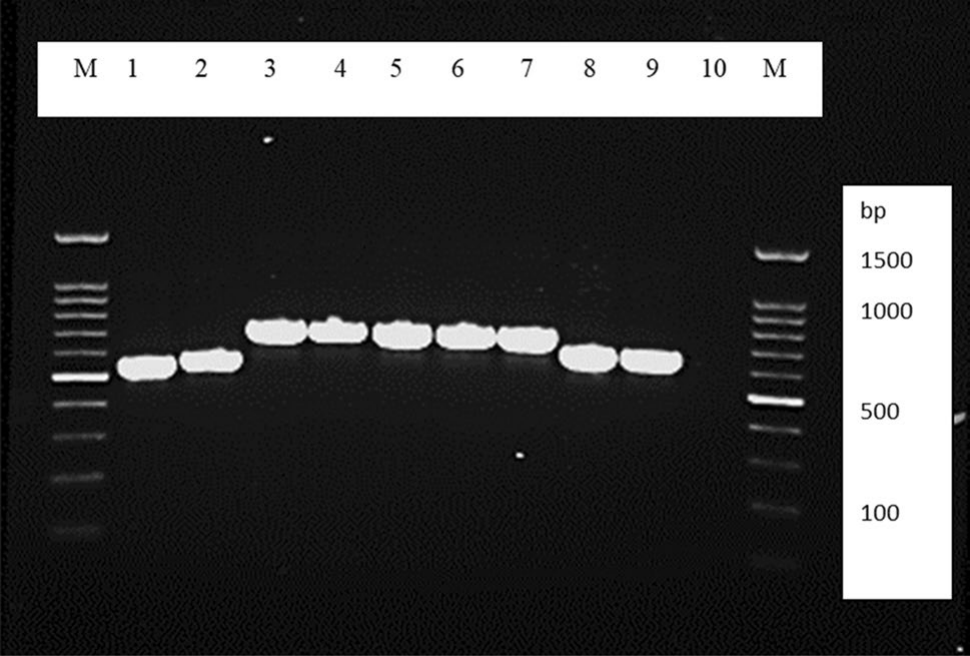
Representative agarose gel electrophoresis of PCR products Lane 1: *Cladosporium halatolerans*; Lane 2: *Alternaria porri*; Lanes 3–6: *Ochroconis musae;* Lanes 7: *Ochroconis constricta;* Lane 8: *Exophiala mesophila;* Lane 9: positive control*;* Lane 10: negative control (D.W.); and Lane M is the 100-bp molecular size marker.

Table 3 summarizes the identification and quantification of black mold, yeast, and yeast-like species of fungi obtained through conventional and molecular methodologies. The most commonly isolated black mold genus was *Cladosporium* spp. (10.7%) followed by Ochroconis spp. (9%), which was the second most frequently detected genus in tested building WMs. As depicted in Fig. 3, the ITS and -tubulin regions of extracted DNA were successfully amplified and the generated sequences from this study have been deposited in the GenBank database.

### The potential for fungal agents to transfer from contaminated WMs to autoclaved socks

This investigation aimed to explore whether fungal agents, originally harbored within WMs that were previously identified as contaminated, could be transferred to sterilized socks during the washing process. Remarkably, all ten of the autoclaved socks used in this study (n = 10, 100%) tested positive for fungal culture following their laundering in the contaminated WMs. This unequivocal finding underscores the capacity of fungal agents to not only survive within the WM environment but also to contaminate laundered textiles.

Furthermore, it is noteworthy that the fungal species recovered from the laundered socks exhibited a significant degree of similarity (70% of the time) to those previously isolated from the contaminated WMs.

## Discussion

In the present day, people spend a significant amount of their time inside buildings, whether it's in their homes, offices, schools, or other establishments. The cleanliness and sanitation of these buildings are paramount for maintaining the health and well-being of individuals. Washing machines are essential appliances that provide an ideal environment for microbial growth^4,8^. They provide a warm and moist environment, which can promote the growth of microbes such as bacteria and fungi. Additionally, residues from detergents and fabric softeners used in WMs can accumulate on various surfaces, including the drum, seals, and detergent drawers. These residues may contain organic matter that serves as a nutrient source for microbes, facilitating their growth. However, when a machine remains unused for extended periods without proper cleaning or maintenance, it becomes susceptible to microbial colonization^7^. Another crucial concern is cross-contamination. If contaminated items, such as soiled clothes or towels with visible dirt, are placed inside WMs, potentially harmful microorganisms can enter the WM's environment, leading to further microbial growth^9,10^.

The primary focus of this study was to assess the potential of building WMs as suitable environments for the growth and proliferation of fungi. Remarkably, we observed a high prevalence (82%) of fungal contamination in these WMs, with a wide array of fungal species identified. Among the various components of WMs examined, the inner rubber door seal was found to be the most frequently colonized area, indicating a preference for fungi to thrive in this specific region. This susceptibility can be attributed to a combination of factors. Firstly, its proximity to the WM drum and the extensive use of water during washing and rinsing cycles create a moist and damp environment, which is conducive to fungal growth, including molds and yeasts. Over time, the accumulation of residues from detergents, fabric softeners, and organic matter within the seal's folds provides essential nutrients for fungi. The generation of heat during machine operation, coupled with moisture from the washing process, establishes favorable temperature conditions for fungal proliferation. Additionally, the limited air circulation within this enclosed space contributes to moisture retention, further promoting fungal growth, as fungi tend to flourish in environments with reduced airflow. The porous nature of the rubber seal offers additional surfaces for fungi to attach and form biofilms, rendering them resistant to removal. Furthermore, the seal's direct contact with laundry items, which may carry fungal contaminants, can lead to the easy transfer of fungal agents onto the seal during washing, introducing fungi into the WM's environment. Lastly, the inner rubber door seal often receives less cleaning attention compared to more visible machine parts, allowing fungal growth to persist and potentially thrive.

Among the isolated fungi, mold fungi (80.3%) were predominant, outnumbering yeast (19.7%) strains. Notably, *Aspergillus* species (27.7%) were the most frequently isolated fungi in this study, followed by *Penicillium* genus (11.4%), and the *Cladosporium* genus (10.7%). Additionally, yeast strains including the *Rhodotorula* genus (12.3%), *Candida parapsilosis* (4.8%), and also *C. orthopsilosis*, and *C. lusitania* (each at 0.8%) were identified. These findings are consistent with previous studies that have highlighted the potential for fungal growth and colonization in damp and humid environments^16^. For example, in the 2019 study by Tischner et al.^7^ which involved 61 WMs, 71 positive cultures were isolated, with mold fungi isolates (55%) being more common than yeast isolates (45%), aligning with our study. In the present study, most of the genera and species of fungal agents isolated from WMs are similar to those in other studies. However, due to the influence of various cultural and environmental factors, as well as climatic differences in each geographical region, the percentage of fungal agents isolated in these studies is variable. Our findings strongly suggest a potential health risk associated with fungal contamination in building WMs. Pathogenic fungi have been linked to various respiratory and allergic diseases, contributing to what is commonly referred to as Sick Building Syndrome (SBS), particularly in individuals with compromised immune systems. Consequently, individuals who frequently use WMs may face an increased risk of exposure to potentially harmful fungi^5^.

In this study, the most commonly isolated genus contaminating WMs was *Aspergillus* spp. In contrast, Tischner et al. found that, the *Penicillium* genus was the most common hyalohyphomycete genus in their study^7^. The *Aspergillus* genus is an opportunistic saprophyte with numerous species and its spores are abundant in our surroundings. It is associated with a wide range of clinical manifestations in various parts of the body, including asthma, allergic bronchopulmonary diseases, eye and sinus infections, onychomycosis, aspergilloma, chronic pulmonary aspergillosis, and diseases related to the central nervous system^17,18^. In this study, *A. flavus* complex, *A. fumigatus* complex, *A. niger* complex, *A. terreus* complex, *A. montevidensis, A. tubingensis, A. calidoustus, A. sydowii, A. multicolor, A. protuberus* were isolated. All of these species have the potential to cause diseases in susceptible hosts. Different species of this genus have different inherent drug sensitivity. For instance, *A. terreus* is typically resistant to amphotericin B. Also, *A. calidoustus* species are known to be resistant to both amphotericin B and voriconazole. Notably, both *A. terreus* and *A. calidoustus* were among the isolates in our study^19^. The genus *Penicillium* is an opportunistic saprophyte that is abundant in building environments and produces secondary metabolites including mycotoxins that can contribute to allergic reactions. In this study, we identified *P. chrysogenum*, a species known for its ability to produce various allergenic metabolites. In some instances, this species has been identified as the causative agent of the invasive disease^20^. The genus *Fusarium* is a common soil saprophyte and a plant pathogen known for its capacity to produce mycotoxins^21^. In this study, we isolated *F. prolifratum*, which has been reported as a pathogenic species^22^. *Fusarium solani* is the most common infection-causing species of this genus, which was isolated from WMs in this study. Additionally, we identified a group of fungi in this study referred to as black fungi (19.7%). These fungi are characterized by their visible dark pigmentation on plates, attributed to melanin, and were confirmed through molecular sequencing methods. The black fungi isolated in this study have the potential to cause subcutaneous phaeohyphomycosis diseases, particularly in individuals with compromised immune systems^23,24^. Within the black fungus group, the genus *Ochroconis* (9%) was the most commonly isolated. *Ochroconis* species exhibit heat tolerance and can withstand temperatures up to 45 °C. Some species within this genus, such as *O. mirabilis* and *O. musae* have been known to cause subcutaneous and systemic diseases, particularly affecting the lungs and brain, in individuals with immune system deficiencies^25–27^. Furthermore, among the studied WMs, we isolated the *C. parpsilosis* complex (5.6%). This complex of *Candida* species is known to colonize the surfaces of sanitary equipment, including WMs, dishwashers, and water distribution systems. The *C.parapsilosis* species complex, which includes *C. parapsilosis* sensu stricto, *C. metapsilosis*, and *C. orthopsilosis*, is considered a significant hospital opportunists and ranks as the second most common cause of mycosis in immunocompromised patients^28^. Some microorganisms exhibit superior survival capabilities in challenging environments, such as WMs compared to others. Notably, specific bacteria like *Staphylococcus aureus* and fungi like *Candida* species can persist even in the presence of detergents and high water temperatures. These microorganisms also possess the ability to form biofilms, which can facilitate their entry into the host, particularly through prosthetic materials. This species shows more resistance to antifungal drugs. For instance, *Candida* species, such as *C. glabrata*, demonstrate significantly higher resistance to fluconazole, a commonly used antifungal medication, with resistance levels approximately five times higher than those observed in *C. albicans*^28^. In another study that can only detect yeast species, the most yeast isolated from investigated WMs were *Rhodotrula* genus (66.7%) and *C. parapsilosis* (25.3%)^29^. Previous studies have demonstrated that the transfer of fungal species from WMs to clothes can potentially lead to diseases and skin problems^28,30^.

In our present study, we also found evidence supporting the transfer of fungal contamination from WMs to clothes during the washing process. It is pertinent to highlight that the fungal species identified in the laundered socks bore a substantial resemblance, accounting for 70% of cases, to those previously isolated from the contaminated washing machines (WMs). This suggests a potential link between the fungal populations present in the WM's interior and those colonizing laundered items, in this case, socks. Such a connection raises critical questions regarding the mechanisms and pathways through which these fungal agents traverse from the machine to textiles and underscores the need for further investigation into this phenomenon. The findings of this study carry profound implications for hygiene practices, especially within communal laundry facilities where multiple users may unknowingly share potentially contaminated machines. These microorganisms can be transferred from WMs to clothes through various mechanisms, including direct contact with the interior surfaces of the WMs, aerosolization, and waterborne transfer^5,7,13^.

Our findings emphasize the critical need for regular cleaning and disinfection of building WMs. Establishing proper cleaning protocols is essential to eliminate any accumulated dirt or organic matter that can serve as a nutrient source for fungal growth^6^. Additionally, the use of effective disinfectants against fungi is crucial to eradicate any residual spores or hyphae. To minimize fungal contamination, it is essential to implement appropriate practices such as leaving the WM door open after use to allow for air circulation and using dedicated WM cleaners designed to remove microbial agents. However, further research is warranted to comprehensively assess the extent of the risk posed by these contaminated machines and to develop appropriate preventive measures^4,9^. In conclusion, while the study provides valuable insights into fungal contamination in household WMs, it is essential to acknowledge the limitations related to sample size, restricted geographical location, and potential confounding factors such as cultural, architectural, and economic variables. Addressing these limitations in future research efforts can contribute to a more comprehensive and nuanced understanding of fungal contamination in WM across diverse settings.

## Conclusion

The study highlights the widespread fungal contamination in building washing machines (WMs), with the inner rubber door seal being the most commonly affected area. Fungal agents can transfer from contaminated WMs to laundered textiles, posing potential health risks. Regular cleaning and disinfection of WMs are essential to mitigate this issue. The most frequently identified fungal genera were *Aspergillus* and *Cladosporium*, known to cause health problems, especially in immunocompromised individuals. Enhanced hygiene practices in communal laundry facilities are crucial. Further research is needed to develop effective preventive measures.

## Method and material

### Study design

**Figure 4:**
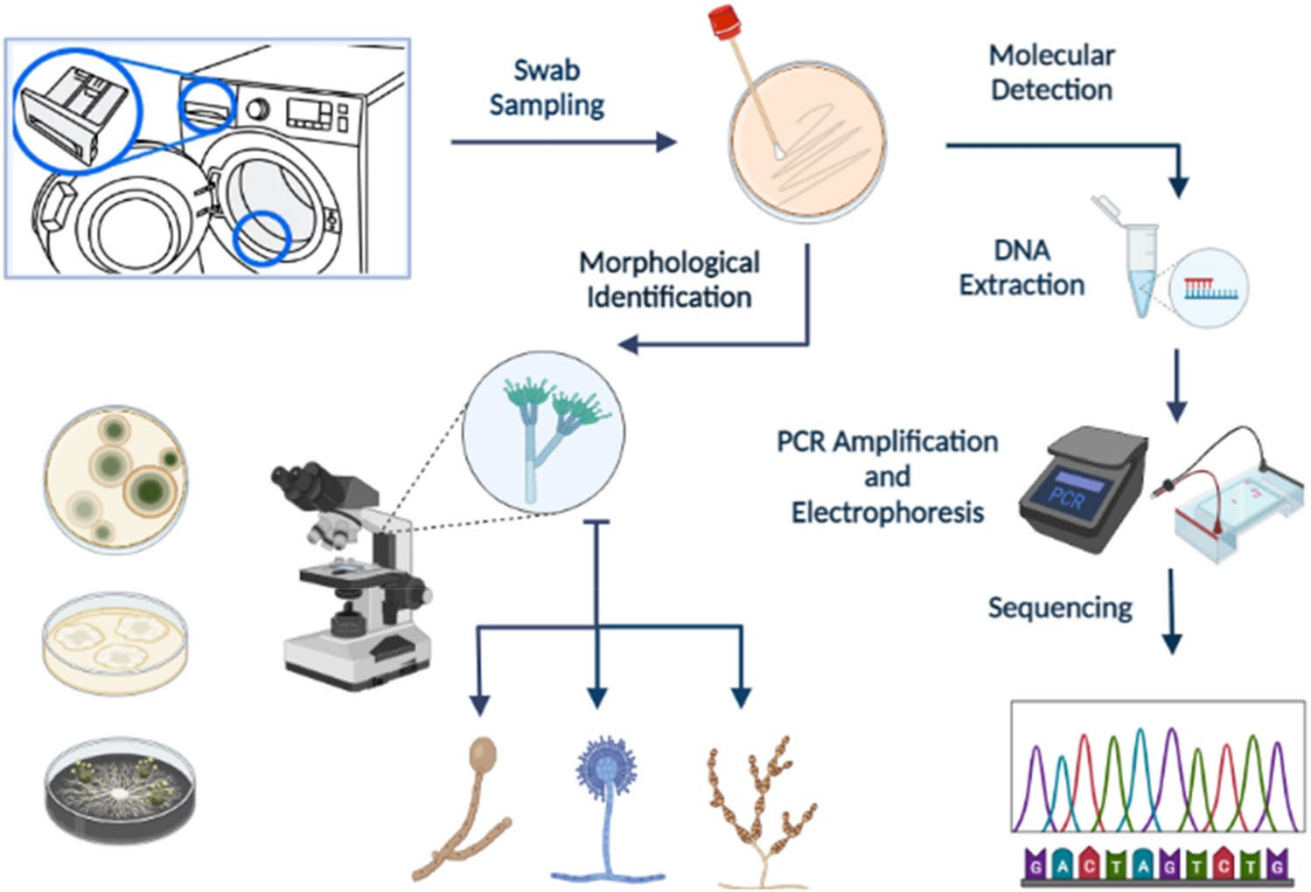
The whole steps of this investigation including, sampling, fungal isolation and identification.

We designed a self-administered questionnaire to assess various usage parameters of the WMs, including user habits, housekeeping conditions, current pigmentation, and the presence and quantity of fungal species. We then distributed these questionnaires and proceeded with WM sampling and device evaluation based on the level of contamination, followed by species identification. The samples were collected from the most contaminated surfaces, as determined by visual assessment, including both the inner and outer parts of the rubber door seal and the detergent drawer. It's noteworthy that all methods described in this study strictly adhered to relevant guidelines and regulations. Additionally, the experimental protocols employed in this study received approval from the Vice-Chancellor for Research of Shiraz University of Medical Sciences. Informed consent was obtained from all participants or their legal guardians prior to their involvement in the study. The whole steps of this investigation are illustrated in Fig. 4.

### Building WMs sampling

Swab samples were collected from 50 household WMs in Shiraz, Iran, during the year 2022. The WMs were sampled at the 3 sites including, the inner and outer parts of the rubber door seal and detergent drawer. These areas are known to be prone to moisture retention and organic residue buildup, creating conditions conducive to fungal growth. The detergent drawer, for instance, can accumulate detergent and fabric softener residues, while the rubber door seal can trap moisture and residues from previous wash cycles. These factors make these sites likely locations for fungal colonization. Mentioned sites of WMs were swapped with pre-moistened sterile cotton swabs in sterile physiological saline (0.9%). The sampling area was ~ 40 cm^2^ for the detergent drawer, ~ 25 cm^2^, and ~ 45 cm^2^ for the rubber door seal, respectively. After that, the swab was transferred to a sterile tube with 2 mL of sterile physiological saline (0.9%) and transported to the medical mycology laboratory. All samples were analyzed within 1 h after sampling^7,8^.

### Fungal isolation and identification of fungi recovered from WMs

Samples from each WMs were inoculated onto two sabouraud dextrose agar (SDA) media containing (0.05%) chloramphenicol and incubated at 32 °C and 25 °C which were monitored for 2 weeks. After the samples were cultured, the pure cultures of the fungi were isolated and stored for further evaluation^7^. The identification of isolated molds and yeasts was based on the morphological (macroscopic-microscopic) features and molecular technique according to sequence analyses.

#### Morphological identification of fungi

**Figure 5:**
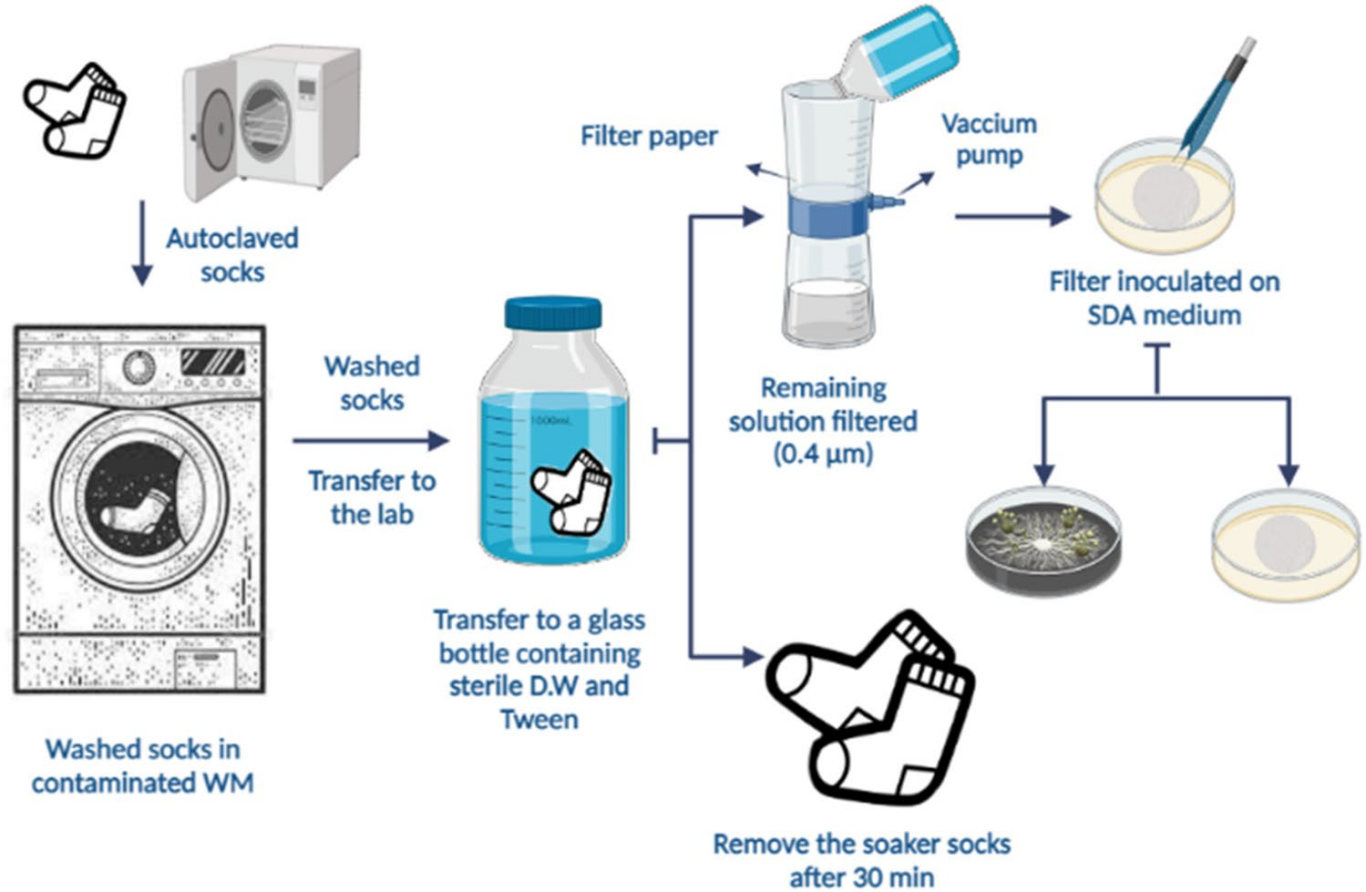
Fungal agents transmission stage from WMs to autoclaved socks.

The gross identification of mold and yeast fungi was performed using mycological laboratory-approved procedures. The development colonies were evaluated for their macroscopic characteristics on SDA, which included the pigment of the colony's surface and underside, shape, structure, and consistency. Subsequently, microscopic features such as size, shape, and blastospores arrangement, produce pseudohyphae or chlamydospores were assessed by using teas mounts and slide culture methods (Fig. 5)^2^.

#### Molecular identification

##### DNA extraction of yeast

Genomic DNA extraction was performed from purified yeast cultures by using the boiling method described by Makimura et al.^31^. Briefly, a small amount of the yeast colony was suspended in 100 µL of lysis buffer (100 mM Tris–HCl, 0.5% sodium dodecyl sulfate (SDS), and 30 mM ethylene diamine tetra acetic acid (EDTA)) and then boiled for 15 min at 100 °C. Therefore potassium acetate solution (2.5 M) was added and kept in an ice box for 1 h. Then it was removed from the cold chain and centrifuged (16,125×*g* for 5 min), and the supernatant was transferred to a new tube. The extracted DNA in the supernatant was washed with ethanol, dried under sterile conditions, and re-suspended in 50 µL of distilled water (DW) for further usage^32^.

##### DNA extraction of mold

Total genomic DNA was extracted from mold fungi by the rapid preparation method. The small portion of mycelium and conidia were grown on SDA at 25 °C, was inoculated to a 500 µl of lysis buffer (400 mM Tris–HCl [pH = 8], 60 mM EDTA pH [pH = 8], 150 mM NaCl, 1% [w/v] SDS), using a sterile applicator for mycelium disruption. After 10 min at room temperature, adding 150 µl of potassium acetate buffer pH [pH = 4.8] (5 M potassium acetate, acetic acid), to remove cell debris, centrifugation was performed) > 10,000*g* for 1 min (. The supernatant solution was transferred to a new tube, centrifuged, and an equal volume of isopropyl alcohol was added. Then, the tube was centrifuged (> 10,000*g* for 2 min), and the supernatant was discarded. The DNA pellet was washed and centrifuged with ethanol (70% (v/v)), then the supernatant was discarded. The air-dried pellet of DNA dissolved in 50 µl of DW and stored at freezer − 20 °C before use^33^.

##### Amplification and sequencing of the ITS region

Yeast species identification was amplified using an internal transcribed spacer (ITS) region with primers ITS1 (5′-TCCGTAGGTGAACCTGCGG-3′) and ITS4 (5′-TCCTCCGCTTATTGATATGC-3′). The PCR amplification was carried out in a final volume of 50 μL containing 5 μL of PCR buffer, 1.5 mM MgCl_2_, 0.8 mM deoxynucleoside triphosphates (dNTPs, 0.2 mM each), 1.2 U of Taq DNA polymerase (Roche, Mannheim, Germany), 0.5 μM of each primer, 5 µL of DNA template, and finally DW. An initial denaturation step at 94 °C for 4 min was followed by 35 cycles of denaturation at 94 °C for 45s, annealing at 64 °C for 1 min, and extending at 72 °C for 1 min. The PCR procedure was followed by agarose gel electrophoresis (1.0%) and stained with ethidium bromide (EB). Then, amplified PCR products were purified and sequenced (Bioneer Company, South Korea). Basic Local Alignment Search Tool (BLAST) was used for comparison with reference patterns in the NCBI/GeneBank database^29,34^.

##### Amplification and sequencing of the β-tubulin regions

The PCR of the genomic DNA product of mold fungi was characterized according to a standard PCR protocol. A fragment of the β-tubulin (βt.) gene was amplified using the primers βt.1 (5′ GGT AAC CAA ATC GGT GCT GCT TTC -3′) and βt.2 (5′- ACC CTC AGT GTA GTG ACC CTT GGC -3′) following the Glass and Donaldson study (20). The PCR amplification was performed in a final volume of 50 μL containing 5 μL of PCR buffer, 1 mM of MgCl_2_, 2.5 mM dNTPs (0.2 mM each), 1.2 U of Taq DNA polymerase (Roche, Mannheim, Germany), 20 ρM each of the primers, 5 µL of DNA template, and eventually, with DW, a final volume (50 µL). An initial denaturing at 94 °C for 5 min was followed by 32 cycles of denaturation at 92 °C for 45 s, annealing at 54 °C for 45 s, and final extension at 72 °C for 1 min. The PCR products were separated on a 1% agarose gel and stained with EB (21, 22). Then, the PCR products were sequenced and analyzed using the Basic Local Alignment Search Tool (BLAST) and matched with sequences in the NCBI/GeneBank data.

### Investigating the possibility of transferring fungal agents from contaminated WMs to autoclaved socks

In the following, the fungal agents transferring from contaminated WMs to the 10 pairs of autoclaved socks during the washing process are examined. According to Fig. 2, after the washing process in the contaminated WMs, washed socks were transferred to the medical mycology laboratory. In the sterile condition under the laminar airflow, the washed socks from the contaminated WMs were transferred to the glass bottle containing 10 ml of sterile DW and 500 ml of Tween 80. After 30 min, the soaking socks were removed from the glass bottle, and the remaining solution was filtered with a sterile membrane filter (0.45 μm, Merck, Millipore) and vacuum pump. After passing the entire solution through the filtration device, the filter is separated and placed on SDA plates, which were incubated at 32 °C and 25 °C for up to 7 days. The growth colonies were evaluated, and mycological identification was documented^35^.

### Statistical analyses

The statistical analysis was performed using SPSS (version 20) software for checking the statistical significance between the fungi growth and sampling sites of building WMs which *p*-values < 0.05 were considered statistically significant.

## Data Availability

The datasets used and/or analyzed during the current study are available from the corresponding author on reasonable request.
